# Association between the intensity of obstructive sleep apnea and skeletal alterations in the face and hyoid bone

**DOI:** 10.1016/j.bjorl.2020.06.008

**Published:** 2020-07-27

**Authors:** Manoela M. Soares, Fabio L. Romano, Franciele V. da Silva Dias, Jaqueline F. de Souza, Leila A. de Almeida, Carolina S. Miura, Carla E. Itikawa, Mirian A. Matsumoto, Wilma T. Anselmo-Lima, Fabiana C.P. Valera

**Affiliations:** a Universidade de São Paulo, Faculdade de Odontologia de Ribeirão Preto, Divisão de Ortodontia, Ribeirão Preto, SP, Brazil; b Universidade de São Paulo, Faculdade de Medicina de Ribeirão Preto, Hospital das Clínicas, Divisão de Fonoaudiologia, Ribeirão Preto, SP, Brazil; c Universidade de São Paulo, Faculdade de Medicina de Ribeirão Preto, Hospital das Clínicas, Departamento de Neurociências, Ribeirão Preto, Brazil; d Universidade de São Paulo, Faculdade de Medicina de Ribeirão Preto, Hospital das Clínicas, Divisão de Otorrinolaringologia, Ribeirão Preto, SP, Brazil

**Keywords:** Obstructive sleep apnea, Child, Hyoid bone, Cephalometry, Craniofacial alterations

## Abstract

**Introduction:**

The association between the intensity of obstructive sleep apnea and skeletal alterations in the face and hyoid bone is still scarcely addressed in the literature.

**Objective:**

To evaluate whether the intensity of obstructive sleep apnea is associated with craniofacial alterations and the position of the hyoid bone in children with mixed dentition.

**Methods:**

76 children aged 7 to 10 years old were examined by otorhinolaryngological evaluation, polysomnography, and orthodontic assessment, including cephalometry. The participants were divided in 3 groups: primary snoring, mild obstructive sleep apnea and moderate to severe obstructive sleep apnea. Cephalometric measures of the face and hyoid bone were assessed. These measures were compared among the different groups by unpaired Student's *t* test. Moreover, these measures were correlated with the patient's obstructive apnea and hypopnea index variable using Pearson's correlation test.

**Results:**

Of the 76 children, 14 belonged to group 1, with primary snoring; 46 to group 2, with mild obstructive sleep apnea; and 16 to group 3, with moderate-severe obstructive sleep apnea. There was no difference between the groups regarding the craniofacial variables. Children with obstructive sleep apnea showed a longer distance from the hyoid bone to the mandibular plane when compared to the primary snoring group (*p* < 0.05). Between the two obstructive sleep apnea subgroups, patients with moderate or severe disease showed significantly shorter horizontal distance between the hyoid bone and the posterior pharyngeal wall (*p* < 0.05), when compared to the groups with mild obstructive sleep apnea. We also observed a significant positive correlation between obstructive apnea and hypopnea index and the distance from the hyoid to the mandibular plane (*p* < 0.05) as well as a significant negative association between obstructive apnea and hypopnea index and the horizontal distance from the hyoid to the posterior pharyngeal wall (*p* < 0.01).

**Conclusion:**

We did not observe any association between obstructive sleep apnea and linear lateral alterations of the face. In contrast, there is a direct association between obstructive sleep apnea severity and the inferior and posterior position of the hyoid bone in children aged 7 to 10 years old.

## Introduction

Childhood obstructive sleep apnea (OSA) is defined by obstructive events that interrupt ventilation and alter the airway architecture during sleep, whether by partial and prolonged or complete and intermittent upper airway obstruction.^1^ It has a prevalence of 1?"4% in the pediatric population,^1^, ^2^, ^3^ and the main associated symptoms are snoring, witnessed apneas, restless sleep and nocturnal enuresis, followed by agitation, inattention or daytime sleepiness.^1^ When persistent, OSA can lead to neurocognitive alterations, changes in neuropsychomotor or weight development, systemic arterial hypertension and cardiac dysfunction.^1^, ^3^

The diagnosis is based on the presence of the above-mentioned symptoms associated with changes in the polysomnography (PSG) examination. The PSG is considered the gold standard for the diagnosis of the disease,^1^, ^3^, ^4^ because it differentiates children with primary snoring (without major ventilatory repercussions) from those with OSA, in addition to stratifying OSA intensity.

According to Kaditis et al.,^3^ craniofacial alterations are among the factors most often associated with OSA in children. This association is evident in syndromic children with deficiencies in the middle third of the face (Apert, Crouzon and Pfeiffer syndromes) and in patients with mandibular hypoplasia (Pierre-Robin and Treacher Collins syndromes). Moreover, less extreme forms may also be present in non-syndromic children. Among the main alterations related to OSA are included maxillary atresia, maxillary and mandibular retrusion and elongated face.^3^, ^5^ Because of this possible association, cephalometry is considered an important exam in the evaluation of patients with OSA.

The association between OSA and craniofacial alterations is best described in adults. In this age group, in addition to the abovementioned alterations, the more inferior and posterior positioning of the hyoid bone is directly related to the presence and intensity of OSA,^6^, ^7^, ^8^ in addition to a greater chance of residual OSA after uvulopalatopharyngoplasty.^9^

There are few studies in the literature that have assessed the position of the hyoid bone and its association to the intensity of apnea in children. Vieira et al.^10^ reported that children with OSA have a more inferior hyoid bone than children in the same age group who are nasal breathers. The authors, however, did not assess whether OSA intensity is associated with these parameters. In a multivariate statistical model, Au et al.^11^ reported that the hyoid inferiorization was associated with a higher risk for OSA, regardless of age, gender or obesity.

Therefore, the aim of the present study was to assess whether there is an association between the severity of OSA and skeletal facial characteristics, in addition to the position of the hyoid bone in children in the school age group, using cephalometry.

## Methods

Children from *Centro do Respirador Bucal* of HCFMRP-USP (Clinics Hospital Ribeirão Preto Medical School ?" University de São Paulo) with sleep-disordered breathing symptoms were evaluated. The following inclusion criteria were considered: children of both genders; with mixed dentition and aged between 7 and 10 years; with respiratory symptoms and high diagnostic suspicion of OSA; patients with any facial or occlusal pattern. The exclusion criteria were the following: children with genetic syndromes and those with a previous history of otorhinolaryngological surgery, orthodontic and/or speech therapy treatment; patients with poor dental condition, or in whom the change of dentition did not allow orthodontic evaluation; patients who had a BMI (body mass index) >95th percentile.

After clarification of the study and the signing of the Term of Assent by the child and the Free and Informed Consent form by the parents/tutors, the participants underwent an otorhinolaryngological clinical evaluation to confirm the respiratory symptoms and their cause. During the clinical evaluation, the BMI was obtained (to confirm that the child was not obese), the degree of hypertrophy of the palatine tonsils at oroscopy (according to the Brodsky Classification),^12^ and the degree of adenoid obstruction in relation to the choana by nasal endoscopy. Children with other causes for respiratory obstruction, in addition to adenotonsillar hypertrophy or allergic rhinitis, were excluded at this stage.

All study participants underwent the PSG exam to confirm the absence or presence of OSA and to stratify its intensity. The parameters of the 2012 American Academy of Sleep Medicine (AASM 2012) were used for that purpose.^13^ The PSG was performed on a BioLogic?(r) digital polygraph and analyzed using the SleepScan Vision?(r) software (version 2.03.05). The evaluated parameters were: electroencephalogram (F3-M2, F4-M1, C3-M2, C4-M1, O1-M2, O2-M1), bilateral electrooculogram (E1-M2 and E2-M2), electrocardiogram (modified V2 lead), superficial electromyogram in the mentalis muscle (2 channels), bilaterally in the masseters and bilaterally in the anterior tibial muscles, position sensor, nasal and oral thermistors (Pro-Tech?(r) air and thermal flow sensor), capnography (“end tidal” CO_2_, Capnochek?(r), Smith Medical) with two channels, plethysmographic straps (Pro-Tech zRIP?(r) respiratory inductance plethysmography), pulse oximeter and microphone.

Unlike adults, where the diagnostic and stratification criteria for OSA are well established, OSA classification and stratification remain a matter of debate in children.^14^ In our center, we use the criteria adapted from the European Respiratory Society Task Force,^3^ which in 2016 considered a diagnostic criterion for OSA when the child had associated symptoms or morbidities and (a) AHI >2; or (b) OAHI >1. Also, according to the same Task Force, children with indexes >5 need more intense care, with a lower rate of spontaneous resolution, and a greater chance of comorbidities.

Thus, as all children in the present study had respiratory symptoms, they were stratified according to the obstructive apnea and hypopnea index (OAHI) as:

- Group 1 (PS ?" Primary Snoring): children with respiratory symptoms and polysomnography confirming the presence of snoring, but with OAHI <1;
- Group 2 (_M_OSA ?" mild OSA): children with OAHI between 1 and 5;
- Group 3 (_MS_OSA ?" moderate to severe OSA): children with OAHI >5.

**Figure 1:**
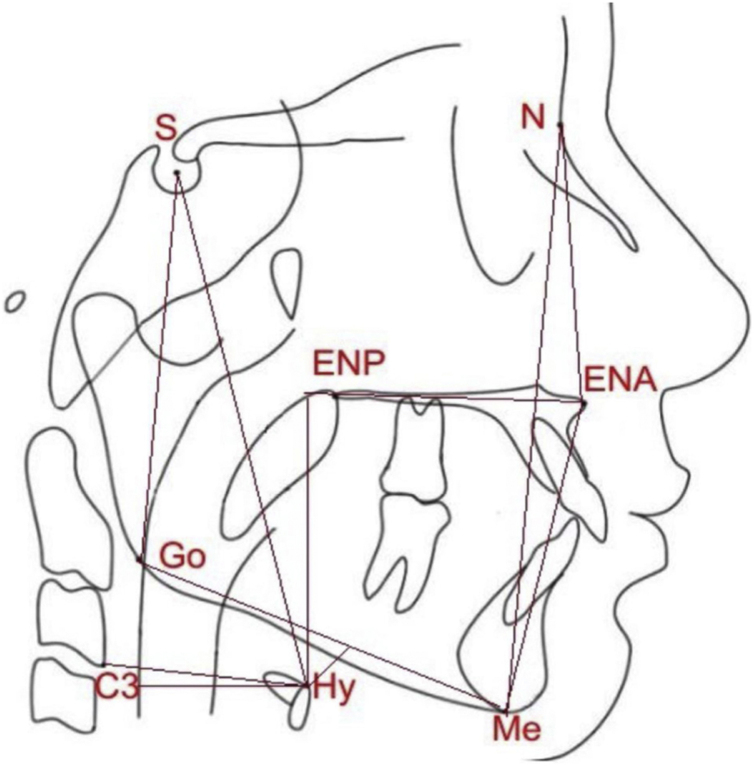
Linear measures analyzed in the present study: N-Me, total anterior facial height; N-ENA, superior anterior facial height; ENA-Me, inferior anterior facial height; S-Go, total posterior facial height; HYS, distance between the hyoid and the base of the skull; HYMP, distance between the hyoid and the mandibular plane (Go-Me); horiz.d-H, measure between the Hy (hyoid) point and the posterior pharyngeal wall; vert.d-H, distance from the Hy point (hyoid) to the palatal plane (ENA-ENP); C3-H, measure from the Hy point (hyoid) to C3 point (cervical column).

The participants were then evaluated with lateral radiography and cephalometric analysis, to confirm craniofacial measures and those related to the position of the hyoid bone (Fig. 1), using the following measures:

N-Me: linear distance representing the total anterior facial height.
N-ENA: linear distance that represents the superior anterior facial height.
ENA-Me: linear distance that represents the inferior anterior facial height.
S-Go: linear distance that represents the total posterior facial height.
HYS: linear distance that represents the distance between the hyoid bone and the base of the skull.
HYMP: linear distance between the hyoid bone and the mandibular plane (Go-Me).
horiz.d-H: linear measure from the Hy (hyoid) point to the posterior pharyngeal wall.
vert.d-H: linear distance from the Hy (hyoid) point to the palatal plane (ENA-ENP).
C3-H: linear measure from Hy (hyoid) point to C3 (cervical column).

Care was taken to ensure that all evaluations were carried out with a maximum interval of 2 months between them.

The project was approved by the Research Ethics Committee of *Clinics Hospital Ribeirão Preto Medical School ?" University de São Paulo* (HCFMRP ?" USP) (Process number 6599/2013).

### Statistical analysis

The data from the otorhinolaryngological and polysomnographic assessments are descriptive and they were used to divide patients into specific groups and, therefore, they were not statistically compared.

To compare the cephalometric variables between the groups, the unpaired Student's *t* test with Welch's correction was applied, considering that the sample distribution passed the normality test, although it was not uniform. Moreover, all cephalometric measures were correlated with the OAHI value, using Pearson's correlation coefficient. The software Graphpad Prism 6.0 was used for all statistical analyses. It was considered statistically different when *p* value was <0.05.

## Results

A total of 76 children participated in the study, with a mean age of 8.2 ± 1.1. The children were divided into: the PS group, consisting of 14 children, 9 boys and 5 girls (mean age 8.2 ± 1.2); and the OSA group, consisting of 62 participants, 34 boys and 28 girls (mean age 8.2 ± 1.3). The OSA group was subdivided into the _M_OSA subgroups, with 46 subjects, 29 boys and 17 girls (mean age 8.3 ± 1.1); and _MS_OSA, with 16 subjects, 5 boys and 11 girls (mean age 8.2 ± 1.4). The mean OAHI index was 0.5 ± 0.2 for the PS group; 2.4 ± 1.1 for the _M_OSA group; and 13.0 ± 8.4 for the _MS_OSA group.

The craniofacial cephalometric measures were initially compared between patients with OSA and the PS group (Table 1), with no difference being observed in any facial linear measure when these two groups were compared. The same result was also observed when comparing the _M_OSA and _MS_OSA groups (Table 2), as there were no differences between the two groups for any craniofacial measure.

**Table 1:** Comparison of craniofacial measures between the OSA and PS groups.

Variables	PS	OSA	*p*	95% CI
N-Me	101.6 ± 1.6	102.0 ± 0.8	0.82	−3.41;4.21
N-ENA	44.3 ± 0.7	44.1 ± 0.4	0.83	−1.95;1.60
ENA-Me	60.2 ± 1.2	60.9 ± 0.6	0.58	−2.02;3.51
S-Go	62.0 ± 1.5	61.4 ± 0.5	0.70	−3.95;2.74

95% CI, 95% confidence interval; PS, primary snoring; OSA, obstructive sleep apnea; N-Me, total anterior facial height; N-ENA, superior anterior facial height; ENA-Me, inferior anterior facial height; S-Go, total posterior facial height.

**Table 2:** Comparison of craniofacial measures between the subgroups _M_OSA *vs.*_MS_OSA.

Variables	_M_OSA	_MS_OSA	*p*	95% CI
N-Me	101.8 ± 1.0	102.5 ± 1.4	0.66	−2.71;4.17
N-ENA	44.0 ± 0.5	44.4 ± 0.8	0.71	−1.63;2.34
ENA-Me	60.7 ± 0.7	61.4 ± 0.9	0.56	−1.67;3.04
S-Go	61.4 ± 0.5	61.8 ± 1.2	0.84	−3.03;2.51

95% CI, 95% confidence interval; _M_OSA, mild obstructive sleep apnea; _MS_OSA, moderate-to-severe obstructive sleep apnea; N-Me, total anterior facial height; N-ENA, superior anterior facial height; ENA-Me, inferior anterior facial height; S-Go, total posterior facial height.

For measures related to the hyoid position, when comparing the PS and the OSA groups, it was observed that children with OSA showed a hyoid position that was significantly more inferior in relation to the mandibular plane (HyMP: 10.9 ± 0.9 for the group PS *vs.* 13.1 ± 0.5 for the OSA group, *p* < 0.05; 95% CI: 0.08; 4.32) (Table 3). When comparing the OSA subgroups (Table 4), children with _MS_OSA had a significantly shorter distance from the hyoid to the posterior pharyngeal wall than those with _M_OSA (horiz.d-H: 24.3 ± 0.4 for _M_OSA and 22.4 ± 0.8 for _MS_OSA *p* < 0.05; 95% CI: −3.76; −0.20).

**Table 3:** Comparison of the cephalometric data of the hyoid bone position between the OSA and PS groups.

Variables	PS	OSA	*p*	95%CI
Hys	86.4 ± 1.3	88.5 ± 0.9	0.18	−1.05;5.25
HyMP	10.9 ± 0.9	13.1 ± 0.5	0.03^a^	0.08;4.32
horiz.d-H	23.7 ± 0.8	24.3 ± 0.6	0.57	−1.51;2.65
vert.d-H	48.6 ± 0.9	50.6 ± 0.7	0.08	−0.27;4.31
C3-H	30.3 ± 0.5	31.2 ± 0.4	0.20	−0.50;2.27

95% CI, 95% confidence interval; PS, primary snoring; OSA, obstructive sleep apnea; HYS, distance between the hyoid and the base of the skull; HYMP, distance between the hyoid and the mandibular plane (Go-Me); horiz.d-H, measure between the Hy (hyoid) point and the posterior pharyngeal wall; vert.d-H, distance from the Hy point (hyoid) to the palatal plane (ENA-ENP); C3-H, measure from Hy point (hyoid) to C3 point (cervical column).

a Value with statistical difference.

**Table 4:** Comparison of cephalometric data of the hyoid bone position between the subgroups _M_OSA *vs.*_MS_OSA.

Variables	_M_OSA	_MS_OSA	*p*	95% CI
Hys	88.4 ± 1.0	88.8 ± 1.9	0.85	−3.96;4.75
HyMP	12.8 ± 0.6	14.2 ± 0.6	0.08	−0.22;3.21
horiz.d-H	24.3 ± 0.4	22.4 ± 0.8	0.03^a^	−3.76;−0.20
vert.d-H	50.5 ± 0.8	50.9 ± 1.4	0.83	−2.98;3.66
C3-H	30.9 ± 0.5	32.0 ± 1.0	0.31	−1.14;3.39

95% CI, 95% confidence interval; _M_OSA, mild obstructive sleep apnea; _MS_OSA, moderate-to-severe obstructive sleep apnea; HYS, distance between the hyoid and the base of the skull; HYMP, distance between the hyoid and the mandibular plane (Go-Me); horiz.d-H, measure between the Hy (hyoid) point and the posterior pharyngeal wall; vert.d-H, distance from the Hy point (hyoid) to the palatal plane (ENA-ENP); C3-H, measure from the Hy point (hyoid) to C3 point (cervical column).

a Value with statistical difference.

**Figure 2:**
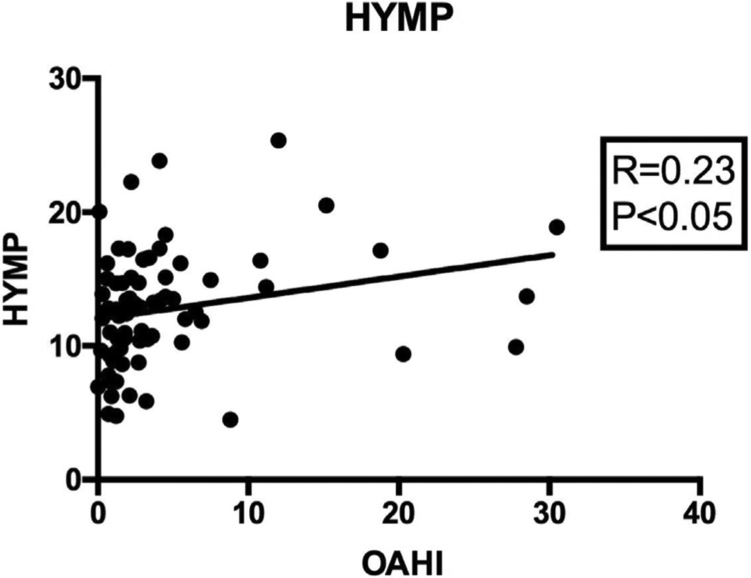
Pearson's correlation between the HYMP cephalometric measure and the OAHI value, showing a positive association between apnea intensity and the distance between the hyoid bone and the mandibular plane.

**Figure 3:**
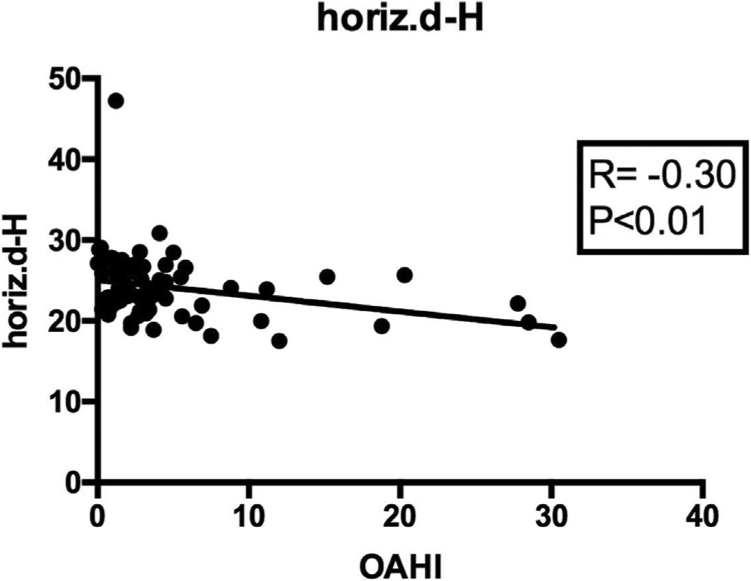
Pearson's correlation between the cephalometric measure horiz.d-H and the OAHI value, showing a negative association between apnea intensity and the distance between the hyoid bone and the posterior pharyngeal wall.

When correlating the OAHI with all cephalometric measures (Table 5), a significantly positive correlation was found between the intensity of apnea and the distance from the hyoid bone to the mandibular plane (HYMP: *R* = 0.236; *p* < 0.05; 95%CI: 0.011; 0.438) (Fig. 2); and a significantly negative correlation was observed between the apnea intensity and the distance from the hyoid to the posterior pharyngeal wall (horiz.d-H: *R* = −0.307; *p* < 0.01; 95%CI: −0.498; −0.087) (Fig. 3). The other measures did not correlate with the OAHI.

**Table 5:** Correlation between the OAHI and each cephalometric measure. Data assessed using Pearson's correlation test.

Variables	*R*	*p*	95%CI
N-Me	0.095	0.411	−0.132;0.314
N-ENA	0.092	0.428	−0.136;0.428
ENA-Me	0.104	0.368	−0.123;0.322
S-Go	0.027	0.815	−0.199;0.251
HYS	0.105	0.363	−0.122;0.323
HYMP	0.236	0.040^a^	0.011;0.438
horiz.d-H	−0.307	0.006^a^	−0.498;−0.087
vert.d-H	0.096	0.397	−0.129;0.317
C3-H	0.138	0.233	−0.090;0352

95%CI, 95% confidence interval; _M_OSA, mild obstructive sleep apnea; _M_SOSA, moderate-to-severe obstructive sleep Apnea; HYS, distance between the hyoid and the base of the skull; HYMP, distance between the hyoid and the mandibular plane (Go-Me); horiz.d-H, measure between the Hy (hyoid) point and the posterior pharyngeal wall; vert.d-H, distance from the Hy point (hyoid) to the palatal plane (ENA-ENP); C3-H, measure from the Hy point (hyoid) to C3 point (cervical column).

a Values with statistical difference.

## Discussion

The association between OSA and craniofacial alterations is better established in adults, and some articles have shown that adult patients with OSA have, in general, maxillary and mandibular retroposition, inferiorization of the hyoid in relation to the mandible or maxilla and increase of the facial height.^6^, ^7^, ^15^, ^16^ Among all cephalometric measures related to OSA in adults, the most consistent ones in the literature, including those supported by meta-analysis studies,^7^ are those related to the more inferior position of the hyoid bone. There is a rationale that the inferiorization of the hyoid is associated with a longer airway and, therefore, more susceptible to pharyngeal collapse. Moreover, it is believed that the inferiorization of the hyoid is associated with less tone in the suprahyoid and base of the tongue muscles, which would potentiate the collapse.

The objective of the present study was to observe whether the facial skeletal characteristics, as well as the position of the hyoid, had any association with the intensity of obstructive sleep apnea in children aged 7?"10 years of age, before the adolescent growth spurt. The main purpose of the present study was to assess whether these bone alterations had already occurred in this age group, or if they developed during adolescence. To confirm the presence of apnea, all children underwent not only the PSG test, considered the “gold standard” for the diagnosis and stratification of OSA,^1^, ^3^ but were also evaluated by a multidisciplinary team, consisting of an otorhinolaryngologist, a neurologist and an orthodontist. In order to contemplate all the study goals within the ethical questions?(tm) aspect, it was decided that the control group would consist of patients with respiratory symptoms, therefore eligible for the polysomnography, but who did not meet the OSA criteria. These children were then considered to have primary snoring.

Although it was not the scope of our analysis, we observed that 45% of the children in the OSA group were girls. The homogeneous distribution between genders in children with OSA has been described in the literature,^1^, ^3^ with no predominance between genders in this age group. Although not significant, there was, however, a predominance of female children in the sample of patients with moderate-to-severe OSA, whereas there was a predominance of male children in the other two groups.

The linear craniofacial measures of facial heights were not statistically different between the groups, either between patients with OSA *versus* those with primary snoring, but also when comparing the _M_OSA with the _MS_OSA groups. That is, in the present group, we found no association between OSA and linear facial measures. This result is in agreement with that observed by Au et al.,^11^ who also did not observe any differences regarding craniofacial measures between the OSA and control groups, or any association between these measures and OSA severity. However, Zicari et al.^17^ observed that children with primary snoring (with a diagnosis based on polysomnography and clinical questionnaires) showed alterations in the angle between the maxilla and the mandible, total divergence and facial growth pattern. Vieira et al.^10^ observed greater total anterior and inferior facial height in the group with OSA compared to those without apnea symptoms, but without polysomnographic confirmation. This difference in results between the studies may have occurred due to divergent selection criteria, or due to the different facial patterns of the patients.

The hyoid bone is important for several functions, including swallowing, and in maintaining the dimensions of the upper airways. Our results show that this bone is more inferior in relation to the mandibular plane in the group with OSA when compared to the primary snoring group, and that patients with moderate-to-severe OSA had a significantly shorter distance from the hyoid bone to the posterior pharyngeal wall when compared to those with mild OSA. The data of the present study are in agreement with those presented by Pirilä-Parkkinen et al.^18^ and Vieira et al.^10^ who also observed a more inferior hyoid in relation to the mandibular plane in patients with OSA. It is noteworthy that the two studies mentioned here also observed a pattern of vertical growth in their patients, which differs from the present study. Moreover, Pirilä-Parkkinen et al.^18^ studied different cephalometric measures than those evaluated here.

We also observed that there is a linear association between the OAHI and some measures: the greater the OAHI, the greater the distance from the hyoid to the mandible, and the shorter the distance from that same bone to the posterior pharyngeal wall. Therefore, the longer and narrower the child's pharynx, the greater the chance that he or she will have obstructive sleep apnea, and the greater its intensity. These results are in agreement with those of Au et al.,^11^ in which the inferiorization of the hyoid was related to OSA intensity in this age group.

Interestingly, although the OAHI has a significant inverse association with the horiz.d-H measure, this association was not observed with another measure of horizontal position of the hyoid, the C3-H. As the C3-H evaluates the distance between the hyoid and the spine posteriorly, while the horiz.d-H evaluates the distance between the hyoid and the posterior pharyngeal wall, one can infer that this finding may be due to the narrowing of the pharynx. Possible causes for this narrowing could be the flaccidity of the hypopharynx muscles, or the accumulation of fat in the same region.

This study has some limitations: the distribution between the groups was not homogeneous, because the vast majority of the children with respiratory sleep disorders showed mild OSA at polysomnography, whereas a smaller number of patients were included in the Primary Snoring or Moderate-to-Severe OSA groups. However, it should be noted that the standard deviation for each of the measures in each group was extremely small, less than 2%, and so the groups can be considered representative. Another bias of the present study was the predominance of girls in the Moderate-to-Severe OSA group, as opposed to the predominance of boys in the other two groups; this bias may have influenced our results.

According to the present findings, we can infer that OSA, in children aged between 7 and 10 years old, does not seem to be related to craniofacial alterations. In contrast, the inferior and posterior position of the hyoid is a measure already observed in this age group, and the persistence of this association can be an important factor for the persistence of OSA in adolescence or even in adulthood.

## Conclusion

Considering these results, one can conclude that in children with mixed dentition, craniofacial alterations do not seem to be consistently associated with the presence of obstructive sleep apnea. On the other hand, the more inferior and posterior position of the hyoid bone, leading to stretching and narrowing of the pharynx, seems to be a predisposing factor for OSA in children.

## Conflicts of interest

The authors declare no conflicts of interest.
